# Current knowledge and “myths” about celiac disease among physicians in the Republic of Kazakhstan: A countrywide cross-sectional study

**DOI:** 10.3389/fpubh.2022.956135

**Published:** 2022-08-12

**Authors:** Aizhan Kozhakhmetova, Serzhan Aidossov, Aissulu Kapassova, Karlygash Borsoldayeva

**Affiliations:** ^1^Biology Department, School of Sciences and Humanities, Nazarbayev University, Nur-Sultan, Kazakhstan; ^2^National Center of Public Health, Nur-Sultan, Kazakhstan; ^3^General Practice Department, Astana Medical University, Nur-Sultan, Kazakhstan; ^4^Administrative Department, Central District Hospital, Talgar, Kazakhstan

**Keywords:** celiac disease, awareness, physicians, country-wide, survey

## Abstract

**Background:**

Celiac disease (CD) is a common genetically predisposed autoimmune condition affecting the gut and other organs. Disease awareness is one of the key components of early case identification. This study aimed to assess awareness about CD among primary care physicians, who are the front-liners in suspecting the diagnosis, and other medical specialists.

**Methods and findings:**

The questionnaire for this survey-based study was created based on the latest international guidelines on CD and included a consent form, 5 general questions (age, gender, etc.), and 10 specific questions concerning CD. Overall, 232 respondents from 13 country provinces (out of 14) and two republican cities were recruited for this study. Of them, 110 (47.4%) were primary care physicians and 122 (52.6%) other medical specialists, including 10 (4.3%) gastroenterologists. A scoring system was used to classify the level of awareness of participants into 3 categories, namely, poor, fair, and good. Analysis of responses revealed poor awareness in 59.4% of physicians, associated with work in republican/province/district/rural/village hospitals (*p* = 0.004), male gender (*p* = 0.006), and age of 40–50 years (*p* = 0.02). The most common “myths” about CD were the following: “symptoms are always obvious in children” or “in adults” (92.5 or 88.4% of respondents, respectively); “genetic mutation *HLA DQ2/DQ8* causes the development of CD in all carriers of the mutation” (51.3%); “CD is a disease of children only” (12.5%); and “is triggered by dairy products” (8.6%). Genotyping of HLA DQ genes has been recommended in case of CD suspicion by every third respondent and was advocated as a “golden standard” confirmatory test by every fifth respondent. A quarter of respondents revealed their incorrect treatment strategies: gluten-free diet for 1 month, dairy-free diet, *Helicobacter pylori* eradication therapy, or responded that did not know how to treat. Overall, 93.5% of respondents expressed intention to learn more about CD, while the rest 6.5% thought that they knew enough, although their knowledge was poor.

**Conclusion:**

This study revealed a poor level of awareness among physicians in Kazakhstan and identified common misconceptions about CD, which potentially could lead to incorrect application of diagnostic tests, delay in diagnosis, and inefficient treatment. Development and implementation of educational programs as well as promotion of self-learning would increase awareness and unravel misconceptions.

## Introduction

Celiac disease (CD) is a genetically predisposed immune-mediated condition caused by gluten ingestion and characterized by gut damage and alteration of many body organs. It has been estimated that 0.6–1% of people worldwide suffer from CD with a significantly increasing annual trend; nevertheless, 80% of cases remain unrecognized. This is due to the fact that the majority of CD cases are silent or atypical and can be masked under other conditions (1–5). It was estimated that an average CD diagnosis delay is 6–10 years, and people attend doctors of different specialties for many years until they get their accurate diagnosis (6). Delay in diagnosis can lead to the development of systemic complications (growth failure and delayed puberty in young children, infertility, osteoporosis, and associated autoimmune and endocrine conditions), sometimes severe ones (enteropathy-associated T-cell lymphoma) (2, 7–9).

In the past, the CD was thought to be more frequent in Northern Europe, Scandinavia (particularly Finland), and Australia and less common in North America and the Middle East. Recent data however show that CD is equally common in all these areas, but apparently is still rare in Sub-Saharan Africa and the Orient– <0.5 % (10, 11). Clinical data from Asian countries (Iran, Turkey, China, and India) suggest a gradually increasing prevalence of CD, too; however, prevalence data from different sources are variable (12–18). This can be explained by many factors: differences in serological methods, screening programs, diagnostic criteria used, as well as varying levels of awareness of symptoms and signs of the disease across the world. Prevalence data in Central Asian countries are very poor or even absent; however, CD in developing countries is reportedly a big public health issue (19–22). There is a lack of epidemiological data for CD in Kazakhstan. The screening study conducted in 2009 among children in Almaty city revealed the disease frequency in children to be 1:262 with the predominance of atypical forms vs. typical (1:5), which were not recognized by out-patient doctors as the CD. Considering the rapid increase of conditions commonly associated with CD in Kazakhstan in the last decades (23–28) as well as an increase in the global prevalence of CD, it is most likely to be involved in this epidemic, and the condition is being hugely underdiagnosed.

There are three pathogenetic components needed for the development of CD: genetic background (HLA DQ2.5 and DQ8 risk genes), an environmental trigger (gliadin), and impaired intestinal permeability (leaky intestinal barrier). The autoimmune reaction in genetically susceptible individuals induces intestinal cell damage (villous atrophy) and causes loss of normal barrier functions (29, 30). The “leaky gut” theory of CD development is based on the disruption of tight junctions that can be caused by different factors: prematurity, radiation, different chemical toxin exposures, etc. It is evident that the composition of the gut microbiome is important for CD development (29–34). Alteration in the balance of the gut microflora has also been reported in some allergies, inflammatory bowel disease, and T1D, while a decrease in microbiome diversity was observed to be associated with autoimmunity (33, 35). In cases of unfavorable exposures, the integrity of the gut is compromised, and antigens become able to pass through the extracellular pathways into the intestinal submucosa, triggering antigen-specific immune responses. Increased intestinal permeability precedes disease and switches on pathological immune responses, causing multi-organ autoimmunity (29, 36).

Disease awareness is one of the key components of early case identification. Knowledge of CD's diverse signs and symptoms and high suspiciousness of physicians are crucial for timely made diagnosis (20, 37–39). Lack of proper knowledge among physicians, especially in primary care, even with the availability of decent equipment, and sensitive and specific diagnostic tests may result in missing the timely diagnosis, wrong utilization of diagnostic methods, and incorrect treatment. This was supported by a recent study that reported the absence of improvement in delays in CD diagnosis in spite of more widely available serological tests (40). In case of low awareness, additional training of primary care physicians on CD has been proven to significantly improve clinical strategies in terms of active recognition of symptoms, testing patients for CD, and proper management of diagnosed patients (41, 42).

In Kazakhstan, the CD identification and diagnosis rate are very low, and the knowledge of primary care physicians, who are the front-liners in CD diagnosis, and other medical specialists, who are frequently engaged in the treatment of associated conditions and complications of CD, has never been assessed. This is the first study in Kazakhstan to identify the level of awareness and reveal pitfalls in the way of timely diagnosis and efficient treatment of CD. The questionnaire used in the survey was available in two official languages of Kazakhstan, namely, Kazakh and Russian. It was created based on the existing clinical guidelines [European Society Pediatric Gastroenterology, Hepatology and Nutrition Guidelines for Diagnosing CD, ESPGHAN, 2020 (43), European Society for the Study of CD Guideline for CD and Other Gluten-Related Disorders, ESsCD, 2019] (44) and was evaluated and confirmed with minor corrections by a small group of practicing gastroenterologists (not study participants) and a translator.

## Materials and methods

### Study design

The online survey was conducted countrywide in order to assess current knowledge about CD among physicians working in different levels of public (primary, secondary, and tertiary) and private medical organizations in Kazakhstan. The sample size was calculated based on the total number of physicians in Kazakhstan (roughly 67 000), confidence interval (6.5%), and confidence level (95%), resulting in sufficient statistical power of the study (above 80%).

Link to the Google form-based questionnaire with the invitation to participate in this study was distributed *via* email to top administrators of 230 outpatient and inpatient hospitals present in the database of the National Center of Public Health (across all 14 provinces of Kazakhstan), which then distributed the link further to all their doctors, independently on specialty, age, experience, or any other factors. The anonymity and volunteering basis of the survey was emphasized. Answers of those study participants who signed the online consent form were automatically collected onto Google Excel datasheet.

### Participants

Overall 246 respondents have agreed to participate in the survey. Of them, 232 respondents represented the target group–practicing physicians, while the other 14 (nurses, receptionists, human resource specialists, administrators, etc.) did not, probably, the link was mistakenly distributed to them; their responses were not included in the analysis. Considering the fact that potentially 6,200 physicians could have received the link, the response rate comprised approximately 4%. However, we were unable to verify if specific top administrators have actually shared the link to doctors of the organization or not.

Of 232 respondents, 154 (66.4%) were from the country's 13 (out of 14) provinces and 74 (31.8%) from 2 republican cities – megapolises – Almaty (former capital) and Nur-Sultan (current capital). Four (1.7%) respondents did not mention their country location (Supplementary Table S1). Majority (151; 65.1%) of respondents have been working in inpatient settings: city hospital (53; 22.8%), university hospital/research center (67; 28.9%), or republican/province/district/rural/village hospital (31; 13.3%). The rest of respondents (81; 34.9%) worked in outpatient settings: public (55; 23.7%) or private (26; 11.2%).

Almost half of the respondents, i.e., 110 (47.4%), were primary care doctors: general practitioners, internists, and pediatricians, and the rest 122 (52.6%) were doctors of narrow specialties including 10 (4.3%) gastroenterologists (children/adult) (Table 1). The answers of gastroenterologists were analyzed separately. The vast majority were women−191 (82.3%). All respondents were categorized into 4 age groups for analysis, namely, under 30 years, 30–40, 40–50, and older than 50 years and into 3 groups based on the duration of work experience, namely, under 5 years, 5–15, and over 15 years of work experience.

**Table 1:** Medical specialty, gender, age, and work experience of respondents participated in the survey.

**Medical specialty**	***N* (%)**	**M/F, n/n (%/%)**	**Age group, years (%)**				Work experience, years (%)*		
			** <30**	**30–40**	**40–50**	**>50**	** <5**	**5–15**	**>15**
**I. Primary care doctors:**	110 (47.4)	16/94 (14.5/85.5)	31 (28.)	24 (21.8)	20 (18.2)	35 (31.8)	40 (36.4)	24 (21.8)	46 (41.8)
General practitioners	52	10/42	22	11	5	14	26	10	16
Internists	28	1/26	4	7	7	9	5	8	14
Pediatricians	28	3/25	5	5	8	10	9	5	14
**II. Doctors of narrow specialties:**	112 (48.3)	25/87 (22.3/77.7)	21 (18.8)	41 (36.6)	26 (23.2)	24 (21.4)	27 (24.1)	41 (36.6)	44 (39.3)
Endocrinologist	10	1/9	2	4	4	-	1	6	3
Neurologist	13	1/12	5	3	3	2	6	1	6
Dermatologist	7	0/7	2	1	2	2	2	1	4
Dentist	9	2/7	3	4	1	1	6	1	2
Other specialists	73	18/55	9	29	16	19	12	32	29
**III. Gastroenterologists:**	10 (4.3)	0/10 (0/100)	3 (30)	3 (30)	1 (10)	3 (30)	2 (22.2)	4 (44.4)	3 (33.3)
Children	1	0/1	-	-	-	1	-	-	1
Adult	8	0/8	3	2	1	2	2	3	2
Children/adult	1	0/1	-	1	-	-	-	1	-
Total (%)	232 (100)	41/191 (17.7/82.3)	55 (23.7)	68 (29.3)	47 (20.2)	62 (26.7)	69 (29.7)	69 (29.7)	93 (40)

^*^One respondent did not mention work experience.

### Ethical consideration

Full ethical approval was received from the Institutional Research Ethics Committee at Nazarbayev University (Nur-Sultan, Kazakhstan), and the study complies with the Declaration of Helsinki Ethical Principles for Medical Research. Permissions and approvals were obtained from each hospital's management. Prior to participation, the participants were provided with detailed information about the study in Kazakh and Russian languages. Digital informed consent was obtained from each participant.

### Questionnaire

The structured questionnaire included a consent form, 5 general questions (questions 1-5: inquiring about age, gender, duration of work experience, specialty, place of work, and country location), and 10 specific questions (questions 6-15) concerning CD (Supplementary material S1).

Questions 6 (*What is CD?*), 7 (*What causes CD?*), 12 (*What examination is necessary to confirm the diagnosis of CD, as a golden standard?*), 13 (*Do you advise close relatives of patients with CD to be examined for CD?*), and 14 (*What is the main treatment for CD?*) allowed one correct answer giving one point for it.

Questions 8 (*For what symptoms and signs can you suspect the presence of CD in an adult (tick all that apply)?* (every correct answer option gave one point, maximum of 10 points), 9 (*For what symptoms and signs can you suspect the presence of CD in a child (tick all that apply)?* (every correct answer option gave one point, maximum of 11 points), and 10 *(Which of the following diseases can be associated with CD (tick all that apply)?* (every correct answer option gave one point, maximum of 12 points) allowed multiple answers to choose from a list of symptoms or conditions, which were all associated with CD. Answer options were organized in a way that allowed respondents to earn points even if they were aware of the most frequently associated conditions and less aware of the rarer ones. Answers “I don't know” or “It is a child disease” gave 0 points. Answers “I don't know since I am a pediatrician” or “I don't know since I treat only adults” gave 0 points; however, none of the respondents used these answer options.

Question 11 (*What examination do you prescribe if you suspect CD in a patient (tick all that apply)? -* did not give points and was designed to analyze physicians' strategies, which could vary depending on the availability and accessibility of diagnostic tests or medical specialists in a specific institution or area.

Question 15 *(Would you like to know more about CD? If so, what information would you like to receive?)* allowed choosing multiple answers and did not give points.

### Data analysis

After checking respondents' answers for completeness and consistency, IBM SPSS 20.0 program was used for data analysis. There were few missing data in the survey responses; however, this was considered during statistical analysis. Normality tests (Kolmogorov-Smirnov and Shapiro-Wilk) were applied, and tests revealed skewness (0.371, standard error 0.161, *p* = 0.009) and kurtosis (-0.056, standard error 0.320, *p* = 0.01) of the data. Mean and standard deviation (SD) were calculated for continuous variables and proportions and percentages for categorical ones. A *p*-value of < 0.05 was considered significant. The chi-squared test was used to determine whether there is a significant difference between the expected frequencies and the observed frequencies in one or more categories.

Along with frequencies of chosen answers, a total score for each respondent was calculated the following way:

*Total score* = ∑*scores for qq. 6, 7, 8, 9, 10, 12, 13, 14* (maximum 38 points)

Mean±SD values for total scores were calculated for each categorical group of respondents and were compared using the *t*-test and analysis of variance (ANOVA).

Based on the total score, the respondent's level of awareness was estimated:

*Level of awareness* = (total score/38)^*^100%

The ordinal scale of levels of awareness ranging from poor (0–39.9%) and fair (40–59.9%) to good (60–100%) knowledge was used and controlled for independent factors (i.e., age, work experience, and gender) using logistic regression.

## Results

### Physicians' responses to the questionnaire

#### Questions about CD etiology

Only 65 (28%) respondents answered correctly that autoimmunity was the underlying mechanism in CD, while the rest of them gave other answers: gene mutation with complete penetrance (119; 51.3%), pathology of large bowel (37; 15.9%), allergic reaction (4; 1.7%), or did not know (5; 2%) (Supplementary Table S2). While equal proportions of primary care physicians (34; 31.5%) and gastroenterologists (3; 30%) have correctly answered this question, the lowest number of correct answers was obtained from physicians of other medical specialties (28; 25%).

The majority (200; 86.2%) of respondents knew that gluten was triggering CD; however, the rest answered that CD was triggered by dairy products (20; 8.6%), gut dysbiosis (3; 1.3%), or an allergy (2; 0.9%); 7 (3%) respondents answered that they did not know.

#### Questions about CD symptoms and syndromes in adults and children

##### CD symptoms and syndromes in adults

Symptoms and syndromes of CD in adults which majority of respondents were aware of frequent abdominal pain/bloating−161 (69.4%), chronic diarrhea/constipation−150 (64.7%), and body underweight−132 (56.9%) (Supplementary Table S2). Irritable bowel syndrome, iron deficient anemia, and chronic fatigue were recognized as possible syndromes present in CD by 122 (52.6%), 110 (47.4%), and 79 (34.1%) respondents, respectively. A quarter of respondents recognized osteoporosis and short stature; 40 (17.2%) agreed that elevation of hepatic ALT and AST levels may be observed; and only 27 (11.6%) respondents agreed that no obvious symptoms may be present in CD in adults. Twenty-nine (12.5%) respondents believed that CD is a disease of children only, while 11 (4.7%) answered that they did not know.

##### CD symptoms and syndromes in children

In children with CD, 176 (77.5%) respondents recognized weight deficiency and decreased muscle mass, 148 (65.2%) chronic diarrhea or constipation, and 143 (63%) frequent abdominal pain (Supplementary Table S2). Iron deficiency anemia, poor appetite, abdominal distention, and irritability or tearfulness were recognized by 102 (44.9%), 93 (41%), 85 (37.4%), and 81 (35.7%) respondents, respectively. The least awareness was about vomiting–it was recognized by 72 (31.7%), short stature−71 (31.3%), frequent colds−35 (15.4%), and absence of obvious symptoms −17 (7.5%) respondents. Eighteen (8%) respondents answered that they did not know.

#### Question about associated conditions and complications of CD

The most commonly recognized conditions associated with CD were osteopenia/osteoporosis–by 93 (40.1%), delayed puberty–by 87 (37.5%), recurrent aphthous stomatitis–by 83 (35.7%), autoimmune gastritis (pernicious anemia)–by 80 (34.5%), and hypoplasia of tooth enamel–by 79 (34.1%) respondents (Supplementary Table S2). A smaller number of respondents recognized infertility−63 (27.2%), autoimmune thyroiditis−59 (25.4%), and immunoglobulin A deficiency−52 (22.94%). The lowest awareness was about type 1 diabetes, dermatitis herpetiformis/psoriasis, peripheral neuropathy/ataxia/epilepsy, and Down and Turner syndromes−46 (19.8%), 45 (19.4%), 34 (14.7%), and 26 (11.2%), respectively. Thirty-four (14.7%) respondents answered “I do not know.”

To assess awareness of directly involved narrow specialists on specific complications and associated conditions, a stratified analysis was performed. It showed that among endocrinologists (*n* = 10), only 6 (60%) recognized delayed sexual development, 5 (50%) autoimmune thyroiditis, 3 (30%) infertility, and 1 (10%) type 1 diabetes as conditions frequently associated with CD. Among dermatologists (*n* = 7), only 2 (28.6%) recognized dermatitis herpetiformis; among neurologists (*n* = 13), only 5 (38.5) recognized peripheral neuropathy, ataxia, and epilepsy; among dentists (*n* = 9), only 2 (22.2%) recognized hypoplasia of tooth enamel or recurrent aphthous stomatitis.

#### Questions about diagnostic tests used in case of CD Suspicion, “golden standard” examination for diagnosis confirmation, and familial susceptibility

The majority of respondents (105; 45.2%) have chosen gastroduodenoscopy with small intestinal biopsy as a first diagnostic examination used in case of CD suspicion. Blood tests for coeliac-specific autoantibodies against tissue transglutaminase (TGA), anti-gliadin antibodies (AGAs), and anti-endomysial antibodies (EMAs) were chosen by 95 (40.9%), 71 (30.6%), and 39 (16.8%) respondents, respectively (Supplementary Table S3). Genotyping of HLA *DQ2/DQ8* genes would be recommended by 83 (35.8%) physicians and fecal fat test, stomach examination, or ultrasound examination of the pancreas by 104 (44.8%), 52 (22.4%), and 30 (12.9%), respectively. Only 39 (17.5%) doctors would refer to gastroenterologists; 16 (6.9%) would refer to endocrinologists, and some doctors (18; 7.7%) would advise a temporary gluten-free diet (GFD) straight away. The rest 19 (8.2%) respondents answered that they did not know.

As a “golden standard” examination for CD diagnosis confirmation, 59 (25.8%) respondents would recommend gastroduodenoscopy with small intestinal biopsy, 56 (24.5%) blood test for TGA, and 47 (20.5%) genotyping of HLA *DQ2/DQ8* genes. AGA and EMA tests would be recommended by 33 (14.4%) and 7 (3.1%), respectively. The rest 227 (11.8%) respondents answered that they did not know.

The majority of respondents, 81.8%, answered positively to the question if close relatives of patients with CD needed to be examined for CD.

#### Question about the treatment of CD

As the main treatment of CD, 205 (89.1%) respondents suggested compliance to GFD (75.2%-lifetime diet and 13.9%-diet for 1 month only), while 11 (4.8%) suggested compliance to dairy-free diet, 4 (1.7%) *Helicobacter pylori* eradication, and 10 (4.3%) respondents chose the answer “I don't know.”

Stratified analysis of responses in different medical groups showed that of 10 gastroenterologists, 9 (90%) would administer a life-long GFD, and 1 (10%) a dairy-free diet. Of 110 primary care physicians, 83 (76.1%) would administer a life-long GFD, 16 (14.7%) a 1-month GFD, 6 (5.5%) a dairy-free diet, and 2 (1.8%) *H. pylori* eradication therapy, while the rest 2 (1.8%) answered that they did not know. Of 112 other medical specialists, 81 (73%) would advise life-long GFD, 16 (14.4%) 1-month GFD, 4 (3.6%) a dairy-free diet, 2 (1.8%) *H. pylori* eradication therapy, and 8 (7.2%) did not know the answer.

### Total scores of respondents

The overall mean total score of respondents was 14.7 ± 6.9 (out of maximal 38 points): primary care physicians and narrow medical specialists having an equal amount of scores - 14.4 ± 6.7 and 14.4 ± 6.8, respectively, and gastroenterologists earning higher scores - 20.8 ± 7.8 (Supplementary Table S4).

Stratification of primary care physicians by country's regions has shown the highest total scores in Western Kazakhstan Province - 26.0 ± 6.37 points and the lowest in Northern Kazakhstan province - 6.25 ± 6.44 points (*p* < 0.05). Mean total scores in other provinces varied between 10.5 and 20.3. Nur-Sultan and Almaty cities earned 14.18 ± 3.91 and 13.33 ± 5.24 points, respectively (Supplementary Figure S1).

Narrow medical specialists (excluding gastroenterologists) earned the highest total scores in Atyrau Province (28.0 ± 0), and the lowest in Eastern Kazakhstan (9.6 ± 3.78) (*p* < 0.05). Other regions earned from 10.57 to 22 points. Nur-Sultan and Almaty cities earned 17.78 ± 5.29 and 15.0 ± 7.08 points, respectively. However, a very small number of representatives from each province does not allow us to use the data for conclusions.

### Level of awareness of respondents and its association with place of work, age, and gender

The categorization of the total cores of respondents into three groups of awareness (poor, fair, and good) showed that more than half of primary care physicians (63; 58.9%) had poor knowledge of CD, every third (35; 32.7%) had fair knowledge, and only 9 (8.4%) respondents demonstrated good knowledge of CD (Table 2). Slightly more narrow medical specialists (not considering gastroenterologists) demonstrated good knowledge-15 (13.4%) respondents; however, the majority - 71 (63.4%)-were poorly knowledgeable.

**Table 2:** Level of awareness of respondents.

**Level of awareness**	**All respondents** ***n* (%)**	**Primary care physicians** ***n* (%)**	**Gastroenterologists** ***n* (%)**	**Other medical specialists** ***n* (%)**
Poor (0–39.9%)	136 (59.4)	63 (58.9)	2 (20)	71 (63.4)
Fair (40–59.9%)	65 (28.4)	35 (32.7)	4 (40)	26 (23.2)
Good (60–100%)	28 (12.2)	9 (8.4)	4 (40)	15 (13.4)
Total	229	107	10	112

Chi-Square test p = 0.012.

Among inpatient medical specialists, respondents working in city hospitals, university hospitals, or research centers have demonstrated higher levels of awareness (40.74 ± 18.81%, 95% CI 3.93–20.01) than those working in republican/province/district/rural/village hospitals (28.76 ± 17.63%, 95% CI 3.91–20.03) (*p* = 0.004).

Respondents from the eldest age group 4 (aged over 50 years) had the highest level of awareness (42.32 ± 19.88%, 95% CI 1.41–16.18) when compared with those from age group 1 (under 30 years) (36.94 ± 18.6%, 95% CI 3.8–10.6, *p* = 0.138), age group 2 (aged 30–40 years) (38.0 ± 17.9%, 95% CI 2.3–11.2, *p* = 0.197), and age group 3 (aged 40–50 years) (33.52 ± 16.48%, 95% CI 0.74–17.94) (*p* = 0.02).

Female respondents demonstrated higher levels of awareness (39.86 ± 18.51%, 95% CI 19.18–3.91) when compared with male respondents (28.31 ± 16.14%, 95% CI 19.65–3.44) (*p* = 0.006).

### Interest in additional learning about CD expressed by respondents

The vast majority of respondents (*n* = 217; 93.53%) expressed their intention to learn more about CD in one or more aspects: methods of diagnosis (63.36%), treatment (61.21%), causes of the disease (50.86%), and disease symptoms (37.93%) (Supplementary Table S5). The overall mean level of awareness in these respondents was poor-37.8%. The rest of the respondents (*n* = 15; 6.47%), answered that they knew enough and did not need additional training, although the mean level of awareness among them (*n* = 15) was also poor−39.8%.

## Discussion

According to meta-analysis, the ratio of identified coeliac patients vs. undiagnosed patients is 1:7-8 (2). Awareness of physicians about CD is crucial for the timely identification of cases and proper follow-up. This study revealed poor knowledge about CD in the majority of primary care physicians and narrow specialists who participated in this study. Predictably, physicians from city hospitals, university clinics, or research centers had better knowledge than colleagues from republican/province/district/rural/village hospitals (*p* = 0.004), which potentially could cause heterogeneity of CD diagnosis rates among more and less urbanized areas of the country. Furthermore, female respondents gave better responses than male respondents (*p* = 0.006). According to available reports, CD awareness in Kazakhstan is much worse than in other countries (38, 45), although male physicians, in contrast, were reported to have better knowledge (45).

The most senior doctors (aged over 50 years) showed the best general knowledge (*p* = 0.02), followed by the youngest age group (under 30 years); and the lowest awareness was observed in the middle age group (40–50 years). These results support previous studies (38) and probably could be explained by the extensive clinical experience of senior doctors and fresh scholarly knowledge of the youngest ones, recent graduates. However, the most senior doctors were least aware of atypical or asymptomatic forms of CD, which are the abundant forms (8, 9); this could be attributed to old medical school, teaching CD as an intestinal malabsorptive disorder. The pattern was also observed among senior doctors in other countries (45). Furthermore, awareness of these forms of CD was critically low in all age groups of respondents and varied in ranges of 8–16% for CD in adults and 3–13% for CD in children. Notably, 12% of respondents mistakenly believe that CD is a disease of children only, while physicians should be better alerted to their adult patients, which usually develop atypical CD more often (46).

Half of the respondents believe that *HLA DQ2* and *HLA DQ8* variants of haplotypes (often referred to among medical professionals as “mutations”) have complete penetrance causing the development of CD in all carriers, which is an obvious misconception. Furthermore, a big proportion of physicians (36%) would recommend *HLA DQ2/DQ8* genotyping as a starting point in case of CD suspicion. This clinical practice contradicts current guidelines, recommending the utilization of HLA genotyping only in difficult and unclear cases, mostly for exclusion of the diagnosis, rather than confirmation (43, 44). Meanwhile, for comparison, only around 1% of doctors in India utilize HLA genotyping for CD diagnosis (47). Approximately 9% of physicians think that CD is triggered by dairy products, not by gluten, revealing a lack of understanding of disease pathogenesis.

Not-scored question 11 allowed revealing diagnostic strategies used by Kazakhstani physicians. Early serological biomarkers of CD circulate in the blood of patients prior to clinical manifestation and treatment. While the EMA test has proven to have the highest specificity in CD, it is very expensive; therefore, it is recommended for diagnostic confirmation in difficult cases only. For routine use, the TGA test is recommended as the first diagnostic tool (43, 44, 48–50). AGA test has the lowest accuracy and maybe only used in children under 2 years of age if at all (30, 43). Our results showed that only 41% of surveyed physicians use TGA, while 17 and 30% may utilize EMA and AGA, respectively. This may be due to a lack of awareness of clinical protocols or limited accessibility to specific diagnostic tests and needs more detailed investigation.

Small intestinal biopsy is considered a “golden standard” examination for confirmation of CD (although in specific cases in children CD diagnosis can be made without it). Only 26% of physicians knew about it, while another 24 and 20% assigned examinations for TGA and *HLA DQ2/DQ8* variants as “golden standards,” respectively. Also, physicians tend to refer patients to additional examinations (i.e., stomach examination and ultrasound examination of the pancreas) that are not informative in case of CD suspicion, or to an appointment with an endocrinologist, which is not a correct clinical practice as well and results in delays in diagnosis.

Although the majority (75%) of physicians were aware of life-long GFD as the only pathogenetic treatment of CD, the rest would inefficiently treat the condition with a temporary GFD, a dairy-free diet, or *H. pylori* eradication therapy.

Close relatives of patients with CD, as well as individuals with other associated conditions (type 1 diabetes, etc.), are also at an increased risk for developing CD, and targeted screening can significantly improve the rate of identification of CD cases (43, 51–53). The majority (82%) of surveyed Kazakhstani physicians were aware of familial susceptibility and would suggest testing close relatives, which is obviously supported by their high awareness of genetic factors of CD. However, two (20%) gastroenterologists would not suggest testing family members, which is worrisome. Poor awareness of associated conditions (11–20%) resembled a similar situation in other countries (47, 54) and needs more attention. The vast majority of physicians recognized their lack of knowledge and were interested to learn more.

**Figure 1:**
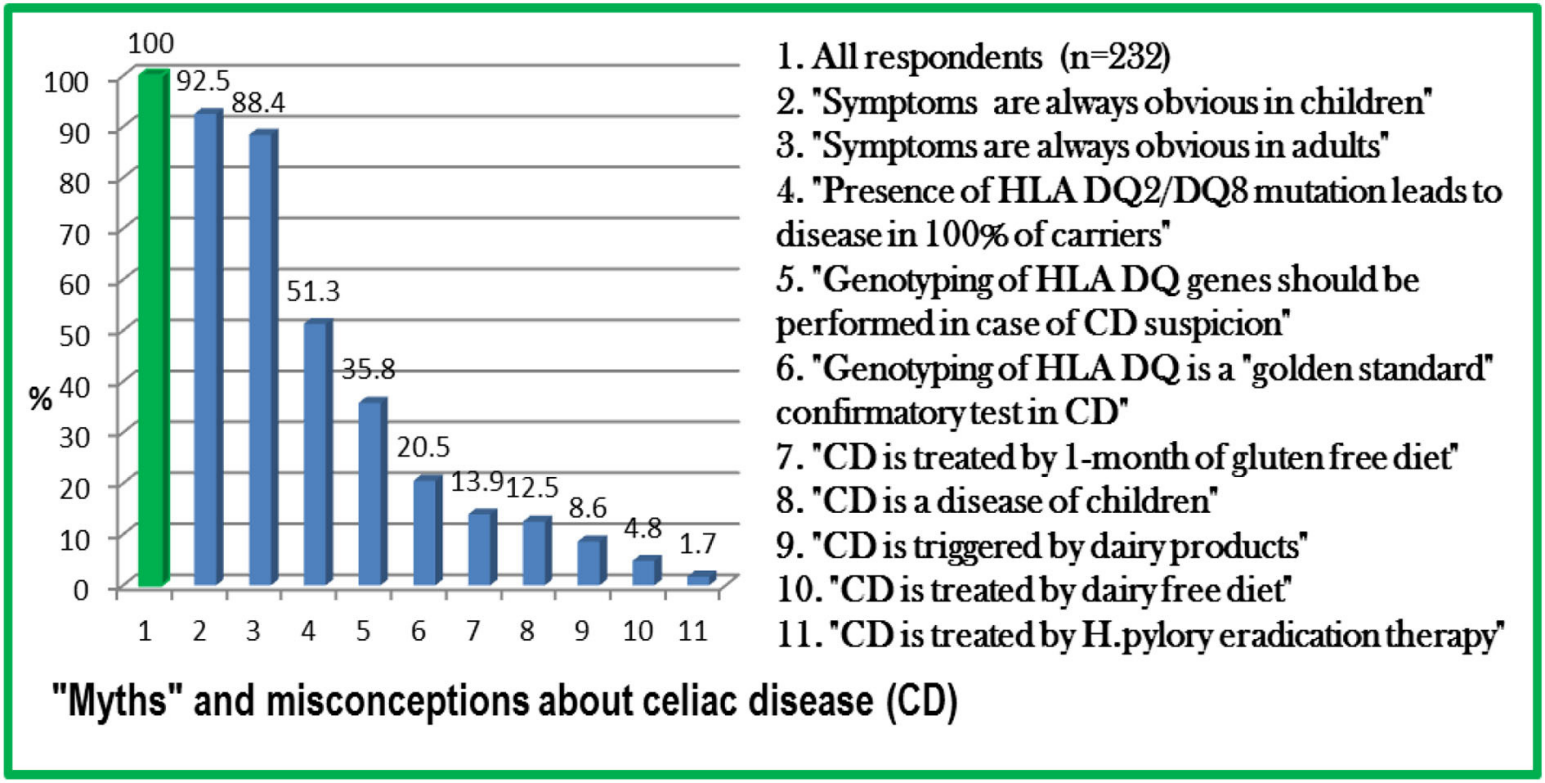
“Myths” and misconceptions about celiac disease (CD) among physicians in Kazakhstan.

All revealed “myths” and misconceptions about CD among physicians are summarized in Figure 1.

This study has several limitations. The first limitation is based on the small number of respondents comprising 0.4% of the total population of doctors in Kazakhstan, and this makes this group less representative of the whole population of physicians. Furthermore, the small number of representatives from different country regions also limits data analysis. It is well known that in spite of the leading position of Kazakhstan in the rate of provision with physicians (3.9 per 1,000 population), there is an irregular distribution of them in provinces as well as a disparity between urbanized and rural areas. Moreover, local factors such as the specialization level of the medical institution, the presence of research centers, and medical schools can modulate the awareness of physicians about CD. Focused surveys targeting specific regions and districts with bigger samples would bring more clarity to the situation in provinces. Another limitation is the online mode of the survey during the ongoing COVID-19 pandemic and the low response rate, which could lead to a selection bias (55–57).

However, to the best of our knowledge, this study is the first of its kind attempting to assess the general knowledge of physicians in Kazakhstan about CD and associated conditions. It gives us a sense of current knowledge and reveals common “myths” and misconceptions that can be present among physicians in this and perhaps in other Post-Soviet countries, which may have a similar medical education system. Although gastroenterologists were not the primary target in the current survey, preliminary results highlight the necessity to assess their knowledge in a separate study with more participants involved.

## Conclusion

Our study revealed an extremely poor level of awareness of different aspects of CD among Kazakhstani physicians. It allowed identifying “myths” about CD, which need to be unraveled. This study has highlighted the urgent need for additional education and/or promotion of self-learning among medical professionals.

## Data availability statement

The raw data supporting the conclusions of this article will be made available by the authors, without undue reservation.

## Ethics statement

The studies involving human participants were reviewed and approved by Institutional Research Ethics Committee at Nazarbayev University (Nur-Sultan, Kazakhstan). Written informed consent for participation was not required for this study in accordance with the national legislation and the institutional requirements.

## Author contributions

AKo: conception, design of study, analysis and interpretation of data, and drafting the manuscript. AKo, SA, AKa, and KB: acquisition of data. All authors have read and approved the final manuscript.

## Funding

Funding (Social Policy Research Grant) for this study was provided to AKo by Nazarbayev University, Republic of Kazakhstan (www.nu.edu.kz). The funders had no role in study design, data collection and analysis, decision to publish, or preparation of the manuscript.

## Conflict of interest

The authors declare that the research was conducted in the absence of any commercial or financial relationships that could be construed as a potential conflict of interest.

## Publisher's note

All claims expressed in this article are solely those of the authors and do not necessarily represent those of their affiliated organizations, or those of the publisher, the editors and the reviewers. Any product that may be evaluated in this article, or claim that may be made by its manufacturer, is not guaranteed or endorsed by the publisher.
