# Supplementary material for: Current knowledge and “myths” about celiac disease among physicians in the Republic of Kazakhstan: A countrywide cross-sectional study
Source: Front Public Health. 2022 Aug 12;10:956135. doi: 10.3389/fpubh.2022.956135 (PMC9411637; doi:10.3389/fpubh.2022.956135)

**Supplementary Material**

**SUPPLEMENTARY APPENDIX 1**

**Questionnaire on Celiac Disease**

**1. Please indicate your gender.**

A. Male

B. Female

**2. Please indicate your age group.**

A. Under 30

B. 30-40 years

C. 40-50 years

D. Over 50

**3. Please indicate your place of work.**

A. District hospital

B. City hospital

C. Province hospital

D. Republican hospital

E. City out-patient public clinic

F. Diagnostic out-patient center

G. Research center hospital

H. Private out-patient clinic

Other______

**4. Please indicate your specialty.**

A. General practitioner (family doctor)

B. Pediatrician

C. Internist

D. Endocrinologist

E. Neurologist

F. Allergologist

G. Dermatologist

H. Dentist

I. Gastroenterologist

Other___________

**5. Please indicate your work experience in the specialty.**

A. Up to 5 years

B. 5-15 years

C. More than 15 years

**6. What is celiac disease?**

A. Allergic disease

B. Autoimmune disease

C. Infectious disease

D. Large bowel disorder

E. Genetic disorder, gene mutation leads to disease in 100% of mutation carriers

**7. What causes celiac disease?**

A. Gluten intolerance

B. Intolerance to dairy products

C. Allergy

D. Gut disbiosis

**8. For what symptoms and signs can you suspect the presence of celiac disease in an adult (tick all that apply)?**

A. Adults do not have celiac disease, it is a childhood disease

B. Chronic diarrhea or constipation

C. Weight deficiency

D. Iron deficiency anemia for unknown reasons

E. Frequent abdominal pain and bloating

F. Short stature

G. Osteoporosis

H. Presence of irritable bowel syndrome

I. The presence of chronic fatigue syndrome

J. Elevated hepatic ALT and AST for unknown reasons

K. No apparent symptoms.

L. I do not know, I am a pediatrician

M. I don't know

**9. For what symptoms and signs can you suspect the presence of celiac disease in a child (tick all that apply)?**

A. Chronic diarrhea or constipation

B. Frequent abdominal pain

C. Big belly

D. Vomiting

E. Weight deficiency, decreased muscle mass

F. Poor appetite

G. Short stature

H. Irritability, tearfulness

I. Iron deficiency anemia for unknown reasons

J. Frequent colds

K. Sometimes no apparent symptoms.

L. I do not know, I only treat adults

M. I don't know

**10. Which of the following diseases can be associated with celiac disease (tick all that apply)?**

A. Delayed sexual development in children

B. Infertility

C. Osteopenia, osteoporosis

D. Immunoglobulin A deficiency

E. Hypoplasia of tooth enamel

F. Recurrent aphthous stomatitis

G. Type 1 diabetes

H. Autoimmune thyroiditis

I. Autoimmune gastritis (pernicious anemia)

J. Herpetiformis dermatitis, psoriasis

K. Down syndrome, Turner syndrome

L. Peripheral neuropathy, ataxia, epilepsy

M. I don't know

**11. What examination do you prescribe if you suspect celiac disease in a patient (tick all that apply)?**

A. Stomach examination (plain fibrogastroduodenoscopy)

B. Fibrogastroduodenoscopy with small intestinal biopsy

C. Ultrasound of the pancreas

D. Examination of feces for fat (coprogram)

E. Blood test for antibodies to tissue transglutaminase

F. Blood test for antibodies to endomysium

G. Blood test for antibodies to gliadin

H. Genotyping of HLA DQ2 and DQ8

I. None, I advise patient to try a gluten-free diet for a while

J. None, I immediately refer to endocrinologist

K. None, I immediately refer to gastroenterologist

L. I don't know

**12. What examination is necessary to confirm the diagnosis of celiac disease (golden standard)?**

A. Antibodies to tissue transglutaminase

B. Antibodies to endomysium

C. Antibodies to gliadin

D. Genotyping for HLA DQ2 and DQ8

E. Fibrogastroduodenoscopy with small intestinal biopsy

F. I don't know

**13. Do you advise close relatives of patients with celiac disease to be examined for celiac disease?**

A. Yes

B. No

**14. What is the main treatment for celiac disease?**

A. Dairy-free diet

B. Antihistamines

C. Gluten-free diet for 1 month

D. H. pylori eradication

E. Lifetime gluten free diet

**15. Would you like to know more about celiac disease? If so, what information would you like to receive?**

A. About the causes of the disease

B. About the symptoms

C. About diagnostic methods

D. About the treatment methods

E. I do not need, I know enough

Other__________

**SUPPLEMENETARY TABLES AND FIGURES**

Table 1. Medical organizations and country location of respondents participated in the survey

| **Country location of respondents** | **Outpatient hospital** | | **Inpatient hospital** | | | **Total**  **n (%)** |
| --- | --- | --- | --- | --- | --- | --- |
|  | **Public**  **n (%)** | **Private**  **n (%)** | **City hospital**  **n (%)** | **University hospital/research center**  **n (%)** | **Republican/province/district/rural/village hospital**  **n (%)** |  |
| **Provinces:** | 27 (17.5) | 13 (8.4) | 25 (16.2) | 64 (41.5) | 25 (16.2) | 154 (66.4) |
| *Akmola* | - | - | - | - | - | **-** |
| *Almaty* | 2 | - | - | 19 | - | 21 (9.0) |
| *Aktobe* | 7 | 5 | 8 | 4 | 5 | 29 (12.5) |
| *Atyrau* | - | - | - | 2 | - | 2 (0.9) |
| *Eastern Kazakhstan* | 1 | 2 | 2 | 1 | 6 | 12 (5.2) |
| *Zhambyl* | - | - | 1 | - | - | 1 (0.4) |
| *Western Kazakhstan* | 5 | - | 1 | - | - | 6 (2.6) |
| *Karaganda* | 3 | 5 | 1 | 4 | 14 | 27 (11.6) |
| *Kostanay* | - | - | 2 | 8 | - | 10 (4.3) |
| *Kyzylorda* | - | - | - | 10 | - | 10 (4.3) |
| *Mangystau* | 2 | - | 1 | 5 | - | 8 (3.4) |
| *Pavlodar* | 4 | 1 | - | 5 | - | 10 (4.3) |
| *Northern Kazakhstan* | 3 | - | 9 | 4 | - | 16 (6.9) |
| *Turkestan* | - | - | - | 2 | - | 2 (0.9) |
| **Republican cities:** | 26 (35.1) | 13 (17.6) | 27 (36.5) | 3 (4.0) | 5 (6.7) | 74 (31.9) |
| *Nur-Sultan* | 3 | 10 | 18 | 1 | 5 | 37 (15.9) |
| *Almaty* | 23 | 3 | 9 | 2 | - | 37 (15.9) |
| Unknown: | 2 | - | 1 | - | 1 | 4 (1.7) |
| **Total n (%)** | 55 (23.7) | 26 (11.2) | 53 (22.8) | 67 (28.9) | 31 (13.3) | 232 (100) |
|  | 81 (34.9) | | 151 (65.1) | | |  |

Table 2. Respondents’ answers for questions regarding etiology, clinical manifestation and associated conditions of celiac disease

| **Questions with answer options** | **All respondents (N=232),**  **n (%)** | **Primary care physicians (N=110),**  **n (%)** | **Gastroenterologists (N=10),**  **n (%)** | **Other medical specialists (N=112),**  **n (%)** |
| --- | --- | --- | --- | --- |
| **6. What is celiac disease?*:** | | | | |
| ***Autoimmune disease*** | 65 (28.0) | 34 (31.5) | 3 (30) | 28 (25) |
| *Genetic disorder due to gene mutation with complete penetrance (leads to disease in 100% of mutation carriers)* | 119 (51.3) | 51 (47.2) | 6 (60) | 62 (55.4) |
| *Large bowel disorder* | 37 (15.9) | 20 (18.4) | 1 (10) | 16 (14.3) |
| *Allergic disease* | 4 (1.7) | 1 (0.9) | - | 3 (2.7) |
| *Infectious disease* | - | - | - | - |
| I don’t know | 5 (2.2) | 2 (1.8) | - | 3 (2.7) |
| **7. What causes celiac disease?** | | | | |
| ***Reaction to gluten*** | 200 (86.2) | 93 (84.5) | 10 (100) | 96 (85.7) |
| *Dairy products intolerance* | 20 (8.6) | 12 (10.9) | - | 8 (7.1) |
| *Gut dysbiosis* | 3 (1.3) | 2 (1.8) | - | 1 (0.9) |
| *Allergy* | 2 (0.9) | 1 (0.9) | - | 1 (0.9) |
| I don’t know | 7 (3) | 1 (0.9) | - | 6 (5.4) |
| **8. For what symptoms and syndromes can you suspect celiac disease in adults?** | | | | |
| ***Frequent abdominal pain and bloating*** | 161 (69.4) | 72 (66.7) | 9 (90.0) | 80 (71.4) |
| ***Chronic diarrhea or constipation*** | 150 (64.7) | 74 (68.5) | 9 (90.0) | 67 (59.8) |
| ***Underweight*** | 132 (56.9) | 65 (60.2) | 8 (80.0) | 59 (52.7) |
| ***Presence of irritable bowel syndrome*** | 122 (52.6) | 53 (49.1) | 5 (50.0) | 64 (57.1) |
| ***Iron deficiency anemia for unknown reasons*** | 110 (47.4) | 53 (49.1) | 9 (90.0) | 48 (42.9) |
| ***Presence of chronic fatigue syndrome*** | 79 (34.1) | 37 (34.3) | 3 (30.0) | 39(34.8) |
| ***Osteoporosis*** | 60 (25.9) | 24 (22.2) | 5 (50.0) | 31 (27.7) |
| ***Short stature*** | 58 (25.0) | 28 (25.9) | 2 (20.0) | 28 (25.0) |
| ***Elevated hepatic ALT and AST for unknown reasons*** | 40 (17.2) | 13 (12.0) | 3 (30.0) | 24 (21.4) |
| ***Obvious symptoms may not be present*** | 27 (11.6) | 9 (8.3) | 3 (30.0) | 15 (13.4) |
| *CD is a disease of children only* | 29 (12.5) | 20 (18.5) | 1 (10.0) | 8 (7.1) |
| I don’t know | 11 (4.7) | 3 (2.8) | - | 8 (7.1) |
| **9. For what symptoms and syndromes can you suspect celiac disease in children?**** | | | | |
| ***Weight deficiency, decreased muscle mass*** | 176 (77.5) | 86 (79.6) | 10 (100.0) | 80 (71.4) |
| ***Chronic diarrhea or constipation*** | 148 (65.2) | 72 (66.7) | 7 (70.0) | 69 (61.6) |
| ***Frequent abdominal pain*** | 143 (63.0) | 68 (63.0) | 7 (70.0) | 68 (60.7) |
| ***Iron deficiency anemia for unknown reasons*** | 102 (44.9) | 49 (45.4) | 9 (90.0) | 44 (39.3) |
| ***Poor appetite*** | 93 (41.0) | 40 (37.0) | 6 (60.0) | 47 (42.0) |
| ***Big belly*** | 85 (37.4) | 42 (38.9) | 5 (50.0) | 38 (33.9) |
| ***Irritability, tearfulness*** | 81 (35.7) | 36 (33.3) | 6 (60.0) | 39 (34.8) |
| ***Vomiting*** | 72 (31.7) | 38 (35.2) | 6 (60.0) | 28 (25.0) |
| ***Short stature*** | 71 (31.3) | 33 (30.6) | 6 (60.0) | 32 (28.6) |
| ***Frequent colds*** | 35 (15.4) | 17 (15.7) | 1 (10.0) | 17 (15.2) |
| ***Obvious symptoms may not be present*** | 17 (7.5) | 4 (3.7) | 4 (40.0) | 9 (8.0) |
| I don’t know | 18 (7.9) | 6 (5.6) | - | 12 (10.7) |
| **10. Which of the following conditions are often associated with celiac disease?** | | | | |
| ***Osteopenia, osteoporosis*** | 93 (40.1) | 43 (39.1) | 3 (30.0) | 47 (42.0) |
| ***Delayed puberty*** | 87 (37.5) | 40 (36.4) | 7 (70.0) | 40 (35.7) |
| ***Recurrent aphthous stomatitis*** | 83 (35.8) | 43 (39.1) | 4 (40.0) | 36 (32.1) |
| ***Autoimmune gastritis (pernicious anemia)*** | 80 (34.5) | 37 (33.6) | 4 (40.0) | 39 (34.8) |
| ***Hypoplasia of tooth enamel*** | 79 (34.1) | 37 (33.6) | 4 (40.0) | 38 (33.9) |
| ***Infertility*** | 63 (27.2) | 29 (26.4) | 5 (50.0) | 29 (25.9) |
| ***Autoimmune thyroiditis*** | 59 (25.4) | 24 (21.8) | 6 (60.0) | 29 (25.9) |
| ***Immunoglobulin A deficiency*** | 52 (22.94) | 27 (24.5) | 4 (40.0) | 21 (18.8) |
| ***Type 1 diabetes*** | 46 (19.8) | 19 (17.3) | 5 (50.0) | 22 (19.6) |
| ***Dermatitis herpetiformis, psoriasis*** | 45 (19.4) | 20 (18.2) | 2 (20.0) | 23 (20.5) |
| ***Peripheral neuropathy, ataxia, epilepsy*** | 34 (14.7) | 15 (13.6) | 3 (30.0) | 16 (14.3) |
| ***Down syndrome, Turner syndrome*** | 26 (11.2) | 14 (12.7) | 1 (10.0) | 11 (9.8) |
| I don’t know | 34 (14.7) | 12 (10.9) | - | 22 (19.6) |
| *out of 230 (99.1%) answers given (2 primary care physicians did not respond)  ** out of 230 answers given (2 physicians of other medical specialties did not respond)  Correct answers are bolded. | | | | |

Table 3. Respondents’ answers for questions regarding diagnostic tests used for diagnosis of celiac disease, follow-up of close relatives and treatment of celiac disease

| **Questions with answer options** | **All respondents (N=232),**  **n (%)** | **Primary care physicians (N=110),**  **n (%)** | **Gastroenterologists (N=10),**  **n (%)** | **Other medical specialists (N=112),**  **n (%)** |
| --- | --- | --- | --- | --- |
| **11. What examination do you prescribe if you suspect celiac disease in a patient?** | | | | |
| ***Blood test for antibodies to tissue transglutaminase*** | 95 (40.9) | 45 (40.9) | 9 (90.0) | 41 (36.6) |
| ***Blood test for antibodies to endomysium*** | 39 (16.8) | 17 (15.4) | 7 (70.0) | 15 (13.4) |
| ***Blood test for antibodies to gliadin*** | 71 (30.6) | 31 (28.2) | 7 (70.0) | 33 (29.5) |
| ***Gastroduodenoscopy with small intestinal biopsy*** | 105 (45.2) | 55 (50.0) | 8 (80.0) | 42 (37.5) |
| *Fecal fat test* | 104 (44.8) | 59 (53.6) | 4 (40.0) | 41 (26.6) |
| *Genotyping of HLA DQ2/DQ8* | 83 (35.8) | 42 (38.2) | 7 (70.0) | 34 (30.4) |
| *Stomach examination (gastroduodenoscopy)* | 52 (22.4) | 30 (27.3) | 2 (20.0) | 20 (17.9) |
| *Ultrasound of the pancreas* | 30 (12.9) | 15 (13.6) | 2 (20.0) | 13 (11.6) |
| *None, I advise patient to try a gluten-free diet for a while* | 18 (7.7) | 7 (6.4) | - | 11 (9.8) |
| *None, I refer to endocrinologist* | 16 (6.9) | 9 (8.2) | - | 7 (6.3) |
| ***None, I refer to gastroenterologist*** | 39 (17.5) | 11(10) | NA | 28 (25) |
| *Other (complete blood count, total IgA, fibrocolonoscopy)* | 3 (1.3) | - | - | 3 (2.7) |
| I don't know | 19 (8.2) | 4 (3.6) | - | 15 (13.4) |
| **12. What examination is necessary to confirm the diagnosis of celiac disease (“golden standard”)?*** | | | | |
| ***Gastroduodenoscopy with small intestinal biopsy*** | 59 (25.8) | 28 (26.2) | 4 (40) | 27 (24.1) |
| *Antibodies to tissue transglutaminase* | 56 (24.5) | 28 (26.2) | 3 (30) | 25 (22.3) |
| *Antibodies to endomysium* | 7 (3.1) | 2 (1.9) | 2 (20) | 3 (2.7) |
| *Antibodies to gliadin* | 33 (14.4) | 14 (13.1) | - | 19 (17) |
| *Genotyping for HLA DQ2 and DQ8* | 47 (20.5) | 28 (26.2) | 1 (10) | 18 (16.1) |
| I don't know | 27 (11.8) | 7 (6.5) | - | 20 (17.9) |
| **13. Do you advise close relatives of patients with celiac disease to be examined for celiac disease?**** | | | | |
| ***Yes*** | 189 (81.8) | 87 (79.8) | 8 (80) | 94 (83.9) |
| *No* | 42 (18.2) | 22 (20.2) | 2 (20) | 18 (16.1) |
| **14. What is the main treatment for celiac disease?***** | | | | |
| ***Lifetime gluten free diet*** | 173 (75.2) | 83 (76.1) | 9 (90.0) | 81 (73.0) |
| *Dairy-free diet* | 11 (4.8) | 6 (5.5) | 1 (10) | 4 (3.6) |
| *Antihistamines* | - | - | - | - |
| *Gluten-free diet for 1 month* | 32 (13.9) | 16 (14.7) | - | 16 (14.4) |
| *H. pylori eradication* | 4 (1.7) | 2 (1.8) |  | 2 (1.8) |
| I don’t know | 10 (4.3) | 2 (1.8) | - | 8 (7.2) |
| * out of 229 answers given (3 primary care physicians did not respond)  ** out of 231 answers given (1 primary care physician did not respond)  *** out of 230 answers given (1 primary care physician and 1 physician of other medical specialty did not respond) | | | | |

Table 4. Mean scores for questions and total scores earned by respondents from different groups

| **Questions** | **All respondents**  **(n=232)** | **Primary care physicians (n=108)** | **Gastroenterologists**  **(n=10)** | **Other medical specialists (n=112)** | p |
| --- | --- | --- | --- | --- | --- |
| **6. What is celiac disease?** (maximum 1 point) | | | | | |
| *Score of respondents: mean±SD (SEM)* | 0.28±0.45 (0.03) | 0.31±0.47 (0.04) | 0.30±0.48 (0.15) | 0.25±0.43 (0.04) | 0.339 |
| **7. What causes celiac disease?** (maximum 1 point) | | | | |  |
| *Score of respondents: mean±SD (SEM)* | 0.86±0.35 (0.02) | 0.85±0.35 (0.03) | 1.00±0 (0) | 0.86±0.35 (0.03) | 0.436 |
| **8. For what symptoms and syndromes can you suspect celiac disease in adults?** (maximum 10 points) | | | | | |
| *Score of respondents: mean±SD (SEM)* | 4.05±2.42  (0.16) | 3.89±2.44  (0.23) | 5.7±2.91 (0.92) | 4.05±2.32 (0.22) | 0.077 |
| **9. For what symptoms and syndromes can you suspect celiac disease in children?** (maximum 11 points) | | | | | |
| *Score of respondents: mean±SD (SEM)* | 4.42±2.56 (0.17) | 4.35±2.43 (0.23) | 6.9±2.64 (0.84) | 4.27±2.57 (0.25) | 0.007 |
| **10. Which of the following conditions are often associated with celiac disease?** (maximum 12 points) | | | | | |
| *Score of respondents: mean±SD (SEM)* | 3.28±2.53 (0.24) | 3.25±2.51 (0.24) | 4.8±2.97 (0.94) | 3.16±2.51 (0.24) | 0.147 |
| **12. What examination is necessary to confirm the diagnosis of celiac disease (“golden standard”)?** (maximum 1 point) | | | | | |
| *Score of respondents: mean±SD (SEM)* | 0.26±0.44 (0.03) | 0.26±0.44 (0.04) | 0.40±0.52 (0.16) | 0.24±0.43 (0.04) | 0.544 |
| **13. Do you advise close relatives of patients with celiac disease to be examined for celiac disease?** (maximum 1 point) | | | | | |
| *Score of respondents: mean±SD (SEM)* | 0.82±0.39 (0.02) | 0.80±0.40 (0.04) | 0.80±0.42 (0.13) | 0.84±0.37 (0.03) | 0.725 |
| **14. What is main treatment for celiac disease?** (maximum 1 point) | | | | | |
| *Score of respondents: mean±SD (SEM)* | 0.75±0.43 (0.029) | 0.76±0.43 (0.041) | 0.90±0.32 (0.10) | 0.73±0.45 (0.04) | 0.471 |
| **Total Score** (maximum 38 points)  *Mean±SD (SEM)* | **14.7±6.9 (0.46)** | **14.4±6.7 (0.65)** | **20.8±7.8 (2.45)** | **14.4±6.8 (0.65)** | 0.016 |

Table 5. Respondents’ answers for the question regarding interest in additional learning about celiac disease

| **Question 15 with answer options** | **All respondents**  **n (%)** | **Primary care physicians**  **n (%)** | **Gastroenterologists**  **n (%)** | **Other medical specialists**  **n (%)** |
| --- | --- | --- | --- | --- |
| **What aspects of CD would you like to know more about?** | | | | |
| *About the causes of the disease* | 118 (50.86) | 62 (57.41) | 3(30) | 53 (47.32) |
| *About the symptoms* | 88 (37.93) | 43 (39.81) | 5 (50) | 40 (35.71) |
| *About diagnostic methods* | 147 (63.36) | 72 (66.67) | 6 (60) | 69 (61.61) |
| *About the treatment methods* | 142 (61.21) | 68 (62.96) | 6 (60) | 68 (60.71) |
| *I do not need, I know enough* | 15 (6.47) | 7 (6.48) | 2 (20) | 6 (5.36) |

Figure 1. Total scores of respondents from different provinces of Kazakhstan


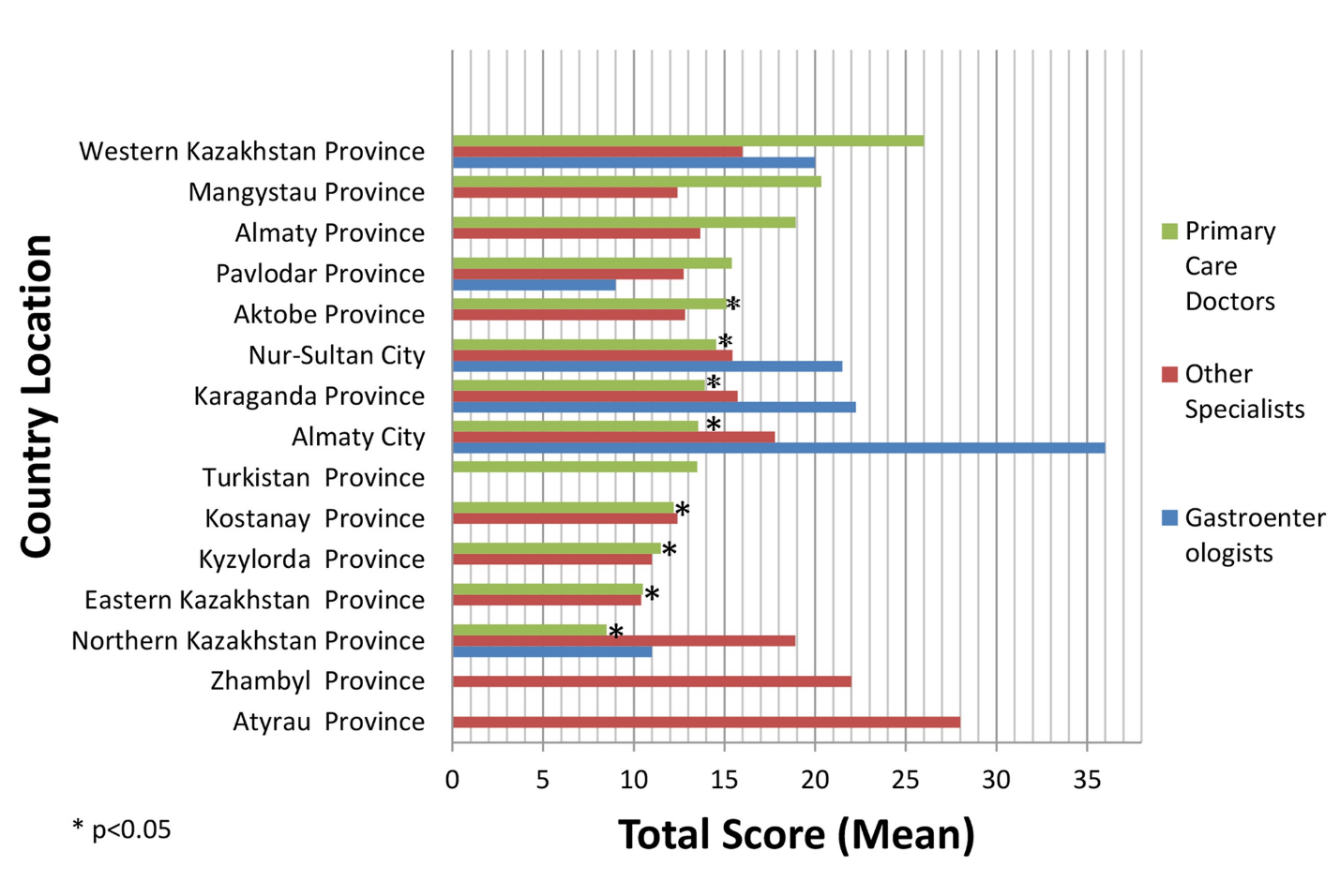

Supplement: Supplementary file 1 [file Data_Sheet_1.docx]
